# A nanostructure of functional targeting epirubicin liposomes dually modified with aminophenyl glucose and cyclic pentapeptide used for brain glioblastoma treatment

**DOI:** 10.18632/oncotarget.5354

**Published:** 2015-09-25

**Authors:** Cheng-Xiang Zhang, Wei-Yu Zhao, Lei Liu, Rui-Jun Ju, Li-Min Mu, Yao Zhao, Fan Zeng, Hong-Jun Xie, Yan Yan, Wan-Liang Lu

**Affiliations:** ^1^ Beijing Key Laboratory of Molecular Pharmaceutics and New Drug System, State Key Laboratory of Natural and Biomimetic Drugs, School of Pharmaceutical Sciences, Peking University, Beijing 100191, China

**Keywords:** Functional targeting epirubicin liposomes, BBB, brain glioblastoma, neovasculatures, mechanism and efficacy

## Abstract

The objectives of the present study were to develop functional targeting epirubicin liposomes for transferring drugs across the blood-brain barrier (BBB), treating glioblastoma, and disabling neovascularization. The studies were performed on glioblastoma cells *in vitro* and on glioblastoma-bearing mice. The results showed that the constructed liposomes had a high encapsulation efficiency for drugs (>95%), suitable particle size (109 nm), and less leakage in the blood component-containing system; were significantly able to be transported across the BBB; and exhibited efficacies in killing glioblastoma cells and in destroying glioblastoma neovasculature *in vitro* and in glioblastoma-bearing mice. The action mechanisms of functional targeting epirubicin liposomes correlated with the following features: the long circulation in the blood system, the ability to be transported across the BBB via glucose transporter-1, and the targeting effects on glioblastoma cells and on the endothelial cells of the glioblastoma neovasculature via the integrin β3 receptor. In conclusion, functional targeting epirubicin liposomes could be used as a potential therapy for treating brain glioblastoma and disabling neovascularization in brain glioblastomas.

## INTRODUCTION

Glioblastoma is a high-grade malignant brain glioma with rich vasculature that support the growth of glioblastoma [1, 2]. Glioblastoma vascularization involves an early co-option of normal brain blood vessels, and the angiogenesis in the tumor region [3, 4]. The walls of the blood vasculature form the blood-brain barrier (BBB), which limits the penetration of chemotherapeutic agents not only into brain parenchyma but also into tumor tissue [5–7]. The treatment of glioblastoma is a comprehensive strategy that mainly uses surgical therapy and is supplemented by radiation therapy and chemotherapy [8, 9]. However, the combined treatment strategy is not able to eliminate all of the glioblastoma cells and cannot destroy the aberrant vasculatures around the glioblastoma tissue. These residual malignant cells and vasculature result in glioblastoma recurrence [10].

In the present work, functional targeting epirubicin liposomes were developed and used for transferring drugs across the BBB, eliminating glioblastoma cells, and destroying the glioblastoma neovasculatures. The anticancer drug epirubicin was encapsulated in the liposomes. The surface of liposomes were modified with a new lipid-glucose conjugate, distearoyl phosphatidylethanolamine polyethylene glycol-4-amino phenyl β-D-glucopyranoside (DSPE-PEG_2000_-Glu), and a lipid-cyclic pentapeptide derivative containing the arginine-glycine-aspartic acid motif (DSPE-PEG_2000_-cRGD) as the targeting molecules.

The BBB is constructed of brain microvascular endothelial cells (BMVECs) with numerous glial cell foot process and neuro cell endings attached to the vascular surface [5]. It is a compact but selective “barrier” that allows endogenous physiological substances to be transported across the BBB in unique ways, including the paracellular aqueous pathway, the transcellular lipophilic pathway, adsorptive transcytosis, receptor-mediated transcytosis and carrier-mediated transcytosis [11]. Nevertheless, the BBB is a hindrance to most exogenous anticancer drugs, leading to the treatment failure of brain tumors with nearly all cytotoxic agents, excluding temozolomide [12].

Glucose transporter 1 (Glut-1) is an endogenous transporter that is expressed on both the luminal and abluminal sides of the BBB [13], acting as a ferry for transferring glucose across the BBB through carrier-mediate transcytosis [14]. 4-Aminophenyl β-D-glucopyranoside (Glu) is a glucose derivative that is transported across the BBB through a glucose transporter [5]. Therefore, in this study, the lipid-glucose conjugate, DSPE-PEG_2000_-Glu, was newly developed as a functional targeting material that was used to modify the liposomes to transport drugs across the BBB.

Recent studies have determined that, compared to those quiescent endothelial cells present on normal tissues, angiogenic endothelial cells in tumor tissues highly express integrin receptors [15–17]. Glioblastoma cells also strongly express integrin receptors [18, 19]. An integrin ligand, such as a cyclic pentapeptide containing a arginine-glycine-aspartic acid motif, is hence used as a targeting molecule by designing a drug delivery system for targeting tumor neovasculature and tumor cells [20–23]. In this study, a thiolated cyclic pentapeptide derivative containing arginine-glycine-aspartic acid motif was conjugated with DSPE-PEG_2000_-maleimide to prepare a targeting material, DSPE-PEG_2000_-cRGD, to modify the liposomes and was used for targeting glioblastoma neovasculature and glioblastoma cells.

As a potent wide-spectrum anticancer drug, epirubicin is able to effectively kill glioblastoma cells *in vitro* but has failed to demonstrate efficacy in the clinical treatment of brain cancer due to the existence of the BBB. Moreover, anthracycline antibiotics are also able to inhibit the growth of endothelial cells [24] and to disturb the neovasculature [25]. Accordingly, epirubicin was selected as the cytotoxic agent in this study and was encapsulated in the functional targeting liposomes to eliminate glioblastoma cells and disabling the neovasculature in the brain tumor region.

Therefore, the objectives of the present study were to develop functional targeting epirubicin liposomes, characterize their efficacy, and determine the mechanisms of transport across the BBB to treat glioblastoma and disable neovascularization.

## RESULTS

### Synthesis of targeting molecules and characterization of the liposomes

**Figure 1:**
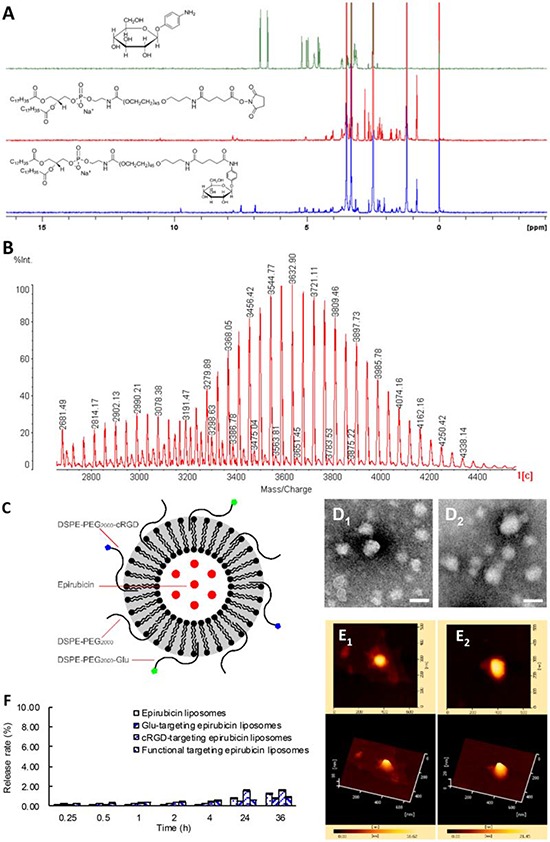
Characterization of targeting molecules and liposomes Notes: **A.** ^1^H NMR spectra of DSPE-PEG_2000_-Glu conjugate. **B.** MALDI-TOF-MS spectra of DSPE-PEG_2000_-cRGD conjugate. **C.** The schematic representation of the functional targeting epirubicin liposomes. **D_1_.** The TEM image of epirubicin liposomes (Bar = 100 nm); **D_2_.** The TEM image of functional targeting epirubicin liposomes (Bar = 100 nm). **E_1_.** The AFM image of epirubicin liposomes; **E_2_.** The AFM image of functional targeting epirubicin liposomes. **F.** The release rates of epirubicin from varying formulations. Data are presented as mean ± standard deviation (*n* = 3).

Fig. 1A and Fig. 1B show the characterizations of the targeting materials. In the ^1^H NMR spectra (Fig. 1A), aromatic proton signals of 4-aminophenyl β-D-glucopyranoside were at 6.00–7.00 ppm, proton signals of polyethylene glycol in DSPE-PEG_2000_ were at ~3.50 ppm, and aromatic proton signals of DSPE-PEG_2000_-Glu shifted from 6.00–7.00 ppm to 7.00–8.00 ppm, indicating a successful synthesis of DSPE-PEG_2000_-Glu. In the MALDI-TOF-MS spectrum (Fig. 1B), the average mass of DSPE-PEG_2000_-cRGD was m/z 3675 and that of DSPE-PEG_2000_-maleimide was m/z 2984. The difference in mass between DSPE-PEG_2000_-cRGD and DSPE-PEG_2000_-maleimide was exactly equal to the difference in mass between c(RGDfK)mpa and 2 mol H, demonstrating a successful synthesis of DSPE-PEG_2000_-cRGD.

Fig. 1C shows a schematic drawing of functional targeting epirubicin liposomes. The surface of the liposome was surrounded by the hydrophilic ends of two targeting conjugates, DSPE-PEG_2000_-Glu and DSPE-PEG_2000_-cRGD. Epirubicin was encapsulated inside the liposome using the ammonium sulfate gradient loading method. A lipophilic fluorescent probe (coumarin 6, DiI or DiR) was encapsulated into a lipid bilayer of the liposome (not shown in the schematic drawing).

Fig. 1D shows TEM images of the epirubicin liposomes (Fig. 1D_1_) and functional targeting epirubicin liposomes (Fig. 1D_2_). Both liposomes were round in shape and approximately 100 nm in diameter. There was no obvious morphologic difference between the epirubicin liposomes and the functional targeting epirubicin liposomes. Fig. 1E shows AFM images of the epirubicin liposomes (Fig. 1E_1_) and functional targeting epirubicin liposomes (Fig. 1E_2_). The appearance and size of the two liposomes were the same as those observed with the TEM.

Fig. 1F illustrates the release rates of epirubicin from varying liposomal formulations. The *in vitro* release rates of epirubicin from all liposomes were below 1% at 2 h, and approximately 2% within 36 h.

Table 1 lists the encapsulation efficiency, particle size, polydispersity index and zeta potential of the epirubicin liposomes, Glu-targeting epirubicin liposomes, cRGD-targeting epirubicin liposomes and functional targeting epirubicin liposomes. In all of the liposomes, the encapsulation efficiency of epirubicin was above 95%. The average particle sizes of the liposomes modified with targeting material(s) were approximately 110 nm with a narrow polydispersity index (~0.20). The epirubicin liposomes modified with targeting material(s) showed a slightly larger size than did the epirubicin liposomes due to the modification with the DSPE-PEG_2000_-Glu or/and DSPE-PEG_2000_-cRGD conjugates. All liposomes were slightly negatively charged.

**Table 1:** Characterization of prepared liposomes

Formulations	Encapsulation efficiency (%)	Particle size (nm)	PDI	Zeta potential (mV)
Epirubicin liposomes	96.88 ± 3.81	97.92 ± 3.15	0.24 ± 0.02	−14.3 ± 1.77
Glu-targeting epirubicin liposomes	97.81 ± 2.70	108.87 ± 3.01	0.20 ± 0.02	−14.6 ± 0.96
cRGD-targeting epirubicin liposomes	98.30 ± 0.91	110.91 ± 19.66	0.21 ± 0.06	−9.63 ± 0.70
Functional targeting epirubicin liposomes	98.68 ± 2.31	108.97 ± 0.67	0.23 ± 0.01	−15.2 ± 0.79

Notes: PDI, polydispersity index; Data are presented as mean ± standard deviation (*n* = 3).

### Cytotoxicity in glioblastoma cells

**Figure 2:**
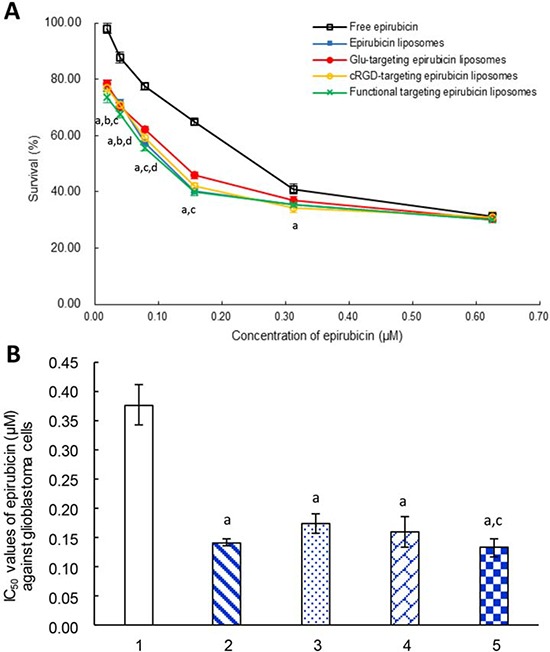
Inhibitory effects to glioblastoma U251 cells Notes: **A.** The survival percentages of glioblastoma cells after treatment with varying formulations at 48 h. **B.** IC_50_ values of epirubicin (μM) against glioblastoma cells after treatment with varying formulations at 48 h. 1. Free epirubicin; 2. Epirubicin liposomes; 3. Glu-targeting epirubicin liposomes; 4. cRGD-targeting epirubicin liposomes; 5. Functional targeting epirubicin liposomes. *p* < 0.05, a, vs. 1; b, vs. 2; c, vs. 3; d, vs. 4. Data are presented as mean ± standard deviation (*n* = 6).

Fig. 2 displays the inhibitory effects on glioblastoma U251 cells after treatment with varying formulations. When treated with a low concentration of epirubicin (<0.6 μM), functional targeting epirubicin liposomes demonstrated significantly stronger killing effects on glioblastoma cells compared to free epirubicin. When treated with a higher concentration of free epirubicin (>0.6 μM), free epirubicin or other liposomal formulations also demonstrated a strong killing effect on glioblastoma cells (Fig. 2A).

The IC_50_ values of varying formulations were calculated to further evaluate the cytotoxicity against glioblastoma U251 cells (Fig. 2B). The IC_50_ values for inhibiting glioblastoma U251 cells were 0.13 ± 0.01 μM for functional targeting epirubicin liposomes, 0.14 ± 0.01 μM for epirubicin liposomes, 0.16 ± 0.03 μM for cRGD-targeting epirubicin liposomes, 0.17 ± 0.02 μM for Glu-targeting epirubicin liposomes, and 0.38 ± 0.03 μM for free epirubicin. Among the varying formulations, functional targeting epirubicin liposomes had the lowest IC_50_ value.

### Targeting effects on BMVECs and on glioblastoma cells

**Figure 3:**
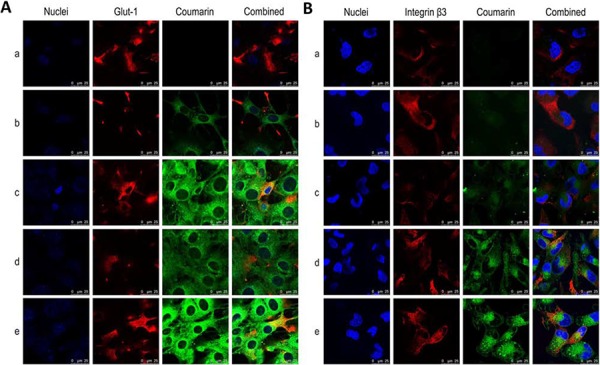
Targeted binding with BMVECs and with glioblastoma cells Notes: **A.** Co-localization of functional targeting coumarin liposomes with Glut-1 on BMVECs. **B.** Co-localization of functional targeting coumarin liposomes with integrin β3 receptors on glioblastoma U251 cells. Nuclei was indicated as blue structure stained by Hoechst 33342; Glut-1 and integrin β3 receptor were indicated as red fluorescence stained by primary and further with secondary antibodies; and liposomes were labeled as green fluorescence with coumarin. The bright-yellow fluorescence was a composite image of green and red fluorescence, and used to indicate the co-localization of liposomes with Glut-1 on the BMVECs or with integrin β3 receptors on the glioblastoma U251 cells. a. Blank control; b. Coumarin liposomes; c. Glu-targeting coumarin liposomes; d. cRGD-targeting coumarin liposomes; e. Functional targeting coumarin liposomes.

Fig. 3 displays the targeted binding of varying fluorescence probe-labeled liposomes with Glut-1 on the BMVECs (Fig. 3A) and with integrin β3 receptors on the glioblastoma U251 cells (Fig. 3A). Glut-1 or integrin β3 receptors were labeled with primary and secondary antibodies and were shown as red fluorescent structures. Liposomes were labeled with coumarin and were shown with green fluorescence. Bright yellow fluorescence is a composite of green and red fluorescence and is used to indicate the targeted binding of the liposomes with Glut-1 on the BMVECs or with integrin β3 receptors on the glioblastoma U251 cells. The results showed that the Glu-targeting coumarin liposomes or the functional coumarin liposomes were bound with the Glut-1 on the BMVECs, in contrast to the coumarin liposomes or cRGD-targeting coumarin liposomes (Fig. 3A), and that the cRGD-targeting coumarin liposomes or the functional targeting coumarin liposomes were bound to the integrin β3 receptors on the glioblastoma U251 cells, in contrast to the coumarin liposomes or Glu-targeting coumarin liposomes (Fig. 3B).

### Uptake and mechanisms of BMVECs and glioblastoma cells

**Figure 4:**
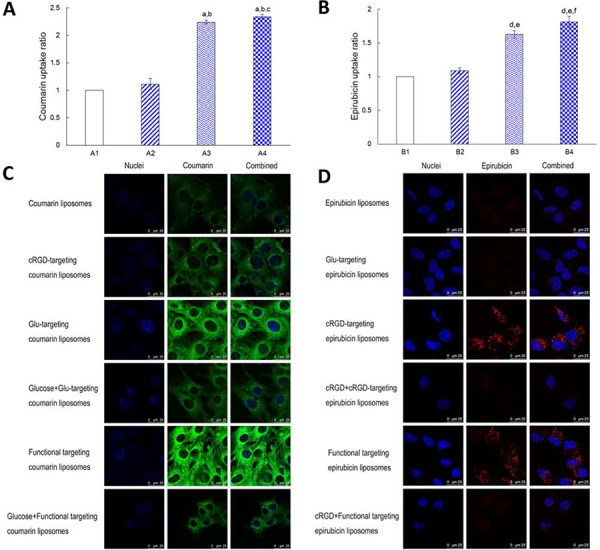
Cellular uptake and competition inhibition effect on the uptake by BMVECs and by glioblastoma cells Notes: **A.** Cellular uptake of varying coumarin labeled liposomes in the BMVECs. **B.** Cellular uptake of varying epirubicin loaded liposomes in glioblastoma cells. A1. Coumarin liposomes; A2. cRGD-targeting coumarin liposomes; A3. Glu-targeting coumarin liposomes; A4. Functional targeting coumarin liposomes. B1. Epirubicin liposomes; B2. Glu-targeting epirubicin liposomes; B3. cRGD-targeting epirubicin liposomes; B4. Functional targeting epirubicin liposomes. **C.** Cellular uptake of DSPE-PEG_2000_-Glu modified liposomes was inhibited in BMVECs by pre-incubation with an excess of free glucose. **D.** Cellular uptake of DSPE-PEG_2000_-cRGD modified liposomes was inhibited in glioblastoma by pre-incubation with an excess of free c(RGDfK)mpa. *p* < 0.05; a, vs. A1; b, vs. A2; c, vs. A3; d, vs. B1; e, vs. B2; f, vs. B3. Data are presented as mean ± standard deviation (*n* = 3).

Fig. 4A illustrates the cellular uptake ratios of varying coumarin-labeled liposomes by BMVECs measured with flow cytometry. The results showed that the rank of cellular uptake ratios was functional targeting coumarin liposomes (2.34 ± 0.04) > Glu-targeting coumarin liposomes (2.24 ± 0.03) > cRGD-targeting coumarin liposomes (1.11 ± 0.10) > coumarin liposomes (with a value of 1 as a reference).

Fig. 4B shows the cellular uptake ratios of varying epirubicin-loaded liposomes by glioblastoma U251 cells measured with flow cytometry. The results showed that the rank of cellular uptake ratios was functional targeting epirubicin liposomes (1.81 ± 0.08) > cRGD-targeting epirubicin liposomes (1.63 ± 0.06) > Glu-targeting epirubicin liposomes (1.09 ± 0.04) > epirubicin liposomes (with a value of 1 as a reference).

Fig. 4C displays the inhibited cellular uptake of varying coumarin-labeled liposomes by BMVECs measured with a confocal laser scanning fluorescent microscope. The results showed that there was an enhanced cellular uptake of DSPE-PEG_2000_-Glu modified liposomes in BMVECs, while this cellular uptake was inhibited by adding excess glucose solution during pre-incubation.

Fig. 4D shows the inhibited cellular uptake of varying epirubicin-loaded liposomes by glioblastoma U251 cells measured with a confocal laser scanning fluorescent microscope. The results showed that there was enhanced cellular uptake of DSPE-PEG_2000_-cRGD modified liposomes in glioblastoma U251 cells, while this cellular uptake was inhibited by adding excess free c(RGDfK)mpa solution during pre-incubation.

### Transport across the BBB and treatment of glioblastoma cells

**Figure 5:**
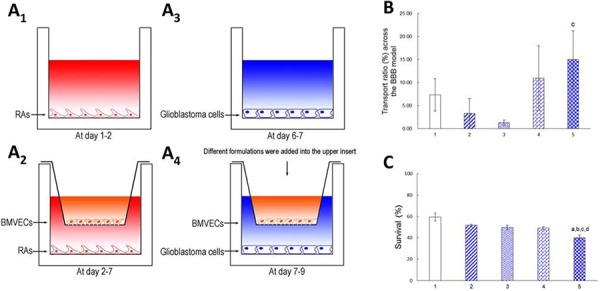
Transporting across the co-cultured BBB model and treating brain glioblastoma cells Notes: **A_1_.** Rat astrocytes (RAs) were seeded on the bottom of the well for 2 days; **A_2_.** Brain microvascular endothelial cells (BMVECs) were then seeded onto the upper insert; **A_3_.** Glioblastoma U251 cells were seeded on the bottom of another well one day before placing the insert; **A_4_.** The co-culture BBB model *in vitro* was established by BMVECs and glioblastoma cells. **B.** Transport ratio of epirubicin across BBB after 3 h incubation. **C.** Dual-targeting effects of functional targeting epirubicin liposomes: crossing the BBB model *in vitro* and then targeted killing glioblastoma cells. 1. Free epirubicin; 2. Epirubicin liposomes; 3. cRGD-targeting epirubicin liposomes; 4. Glu-targeting epirubicin liposomes; 5. Functional targeting epirubicin liposomes. *p* < 0.05; a, vs. 1; b, vs. 2; c, vs. 3; d, vs. 4. Data are presented as mean ± standard deviation (*n* = 3).

Fig. 5A_1_ though Fig. 5A_4_ show schematic drawings of the co-culture BBB model established with BMVECs in the upper insert and with glioblastoma U251 cells in the lower well of a 12-well culture plate, followed by treatment with varying formulations. The integrity of the BBB model was successfully confirmed by measuring the transendothelial electrical resistance (TEER, 165 Ω cm^2^) [26]. The TEER values and liquid levels in the upper insert were monitored during the study, and the results showed that the BBB kept a constant compactness; further, no liquid leakage was observed in the BBB model.

Fig. 5B and Fig. 5C show the transport ratios of the drug across the co-culture BBB model, followed by killing of the glioblastoma U251 cells. After treatment with free epirubicin, epirubicin liposomes, cRGD-targeting epirubicin liposomes, Glu-targeting epirubicin liposomes and functional targeting epirubicin liposomes, the transport ratios of epirubicin at 3 h were 7.33 ± 3.51%, 3.33 ± 3.21%, 1.33 ± 0.58%, 11.00 ± 7.00%, and 15.00 ± 6.24%, respectively (Fig. 5B). Accordingly, after the transport of varying formulations across the BBB, the survival percentages of glioblastoma cells at 48 h were 59.39 ± 3.70%, 51.70 ± 0.95%, 49.71 ± 2.01%, 49.00 ± 1.52%, and 40.13 ± 2.48%, respectively (Fig. 5C).

### Penetration of solid glioblastoma spheroids

**Figure 6:**
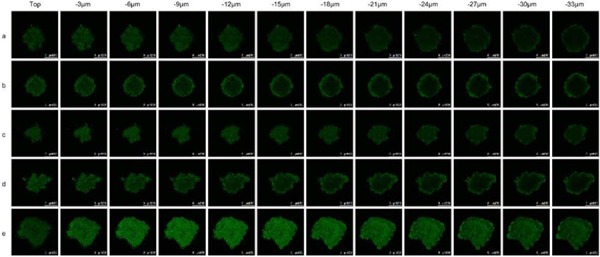
Penetrating ability into solid glioblastoma spheroids Notes: **a.** Free coumarin; **b.** Coumarin liposomes; **c.** Glu-targeting coumarin liposomes; **d.** cRGD-targeting coumarin liposomes; **e.** Functional targeting coumarin liposomes.

Fig. 6 shows the penetration abilities of varying formulations into the glioblastoma U251 spheroids. The results showed that the functional targeting coumarin liposomes had the strongest fluorescent intensities in the core of the glioblastoma spheroids at a depth of 33 μm. In contrast, the fluorescence of other coumarin-labeled liposomes was only distributed in the periphery of the glioblastoma spheroids.

### Inhibiting endothelial cells and destroying neovasculature

**Figure 7:**
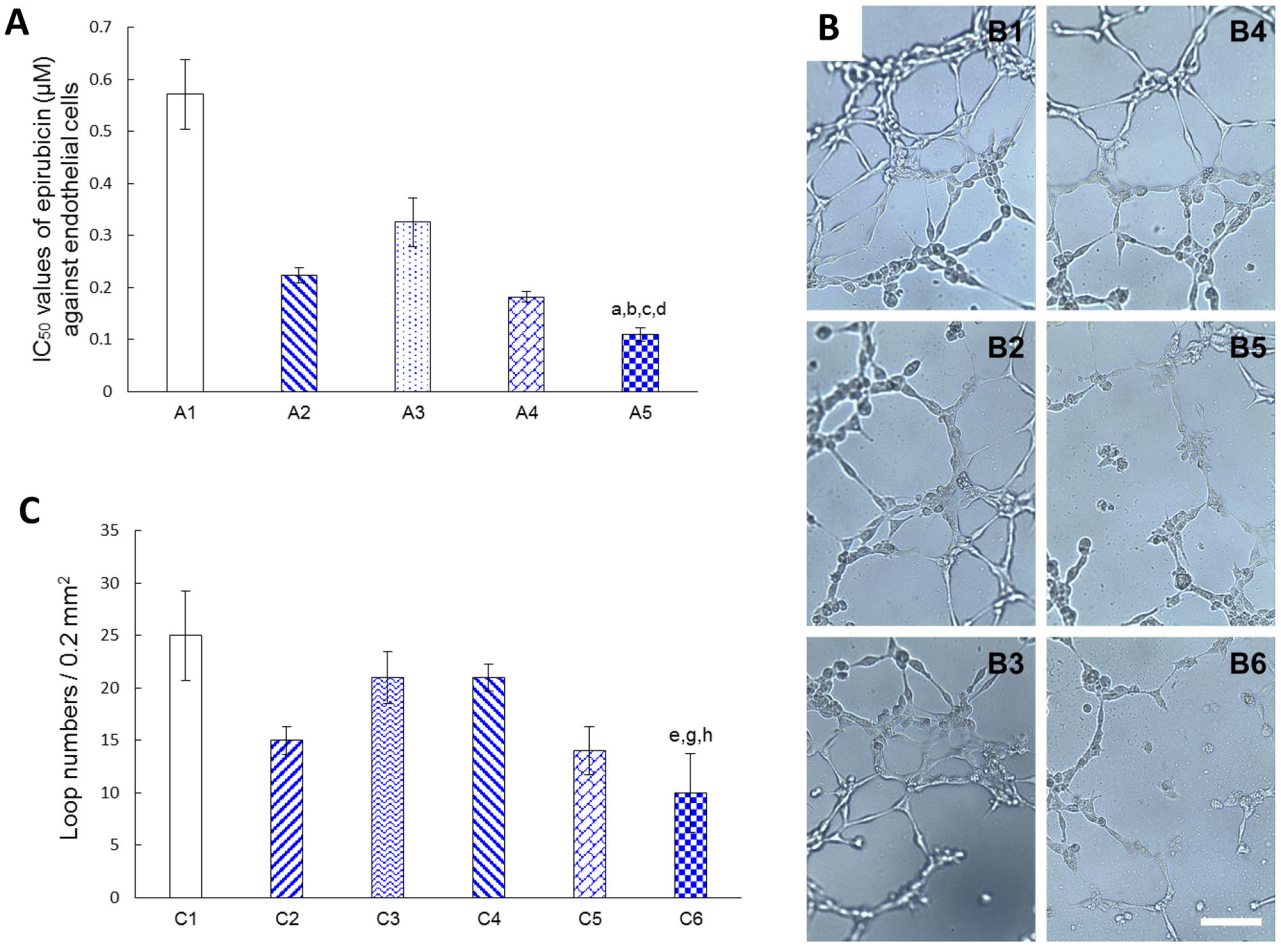
Destroying the neovasculatures *in vitro* Notes: **A.** IC_50_ values of epirubicin (μM) against endothelial cells (EA. hy 926) after treatment with varying formulations at 48 h. A1. Free epirubicin; A2. Epirubicin liposomes; A3. Glu-targeting epirubicin liposomes; A4. cRGD-targeting epirubicin liposomes; A5. Functional targeting epirubicin liposomes. **B.** Destroying effect on the endothelial cells (EA. hy 926) tube formation after treatment with varying formulations at 48 h. B1. Blank control; B2. Free epirubicin; B3. Epirubicin liposomes; B4. Glu-targeting epirubicin liposomes; B5. cRGD-targeting epirubicin liposomes; B6. Functional targeting epirubicin liposomes. **C.** Quantitative analysis of endothelial cells (EA. hy 926) tube formation. C1. Blank control; C2. Free epirubicin; C3. Epirubicin liposomes; C4. Glu-targeting epirubicin liposomes; C5. cRGD-targeting epirubicin liposomes; C6. Functional targeting epirubicin liposomes. *p* < 0.05; a, vs. A1; b, vs. A2; c, vs. A3; d, vs. A4; e, vs. C1; g, vs. C3; h, vs. C4 (Bar = 100 μm). Data are presented as mean ± standard deviation (*n* = 3).

Fig. 7A shows the cytotoxic effects of varying formulations to endothelial cells for mimicking blood vessels in the brain glioblastoma region by measuring the IC_50_ values of the model endothelial cells (EA. hy 926). The results demonstrated that the rank of cytotoxic effects to endothelial cells was functional targeting epirubicin liposomes (0.11 ± 0.01 μM) > cRGD-targeting epirubicin liposomes (0.18 ± 0.01 μM) > epirubicin liposomes (0.22 ± 0.02 μM) > Glu-targeting epirubicin liposomes (0.33 ± 0.05 μM) > free epirubicin (0.57 ± 0.07 μM), suggesting that the functional targeting epirubicin liposomes had the strongest inhibitory effects on endothelial cells.

Fig. 7B indicates the destructive effects on the model neovasculatures after treatment with varying formulations. The model neovasculatures were capillary-like structures formed by endothelial cells (EA. hy 926) and were significantly damaged by the addition of functional targeting epirubicin liposomes compared to the addition of other control formulations.

Fig. 7C displays the numbers of endothelial cell loops as a method of evaluating the above disabling effects on model neovasculatures after treatment with varying formulations. The results showed that the functional targeting epirubicin liposomes or cRGD-targeting epirubicin liposomes significantly reduced the loop numbers compared to other liposomal controls, suggesting a strong disabling effect on neovasculatures.

### Co-localizing with neovasculature and cancer cells in brain glioblastoma-bearing mice

**Figure 8:**
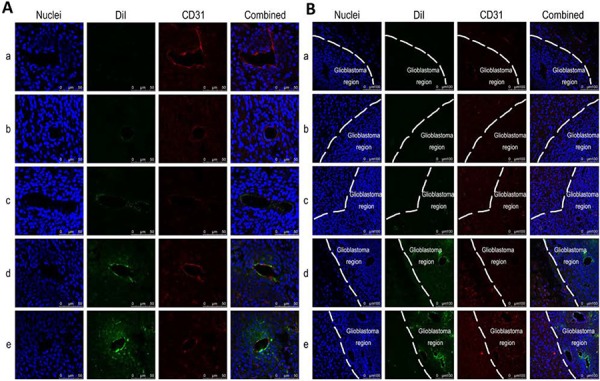
*In vivo* co-localization or targeting binding with neovasculatures and with glioblastoma cells in brain glioblastoma-bearing mice Notes: **A.** Co-localization of functional targeting DiI liposomes with neovasculature in glioblastoma. **B.** Distribution of functional targeting DiI liposomes in brain tissue. Nuclei was indicated as blue structure stained by Hoechst 33342. DiI labeled liposomes were seen as green fluorescence. Glioblastoma neovasculature was indicated as red fluorescence stained by anti-CD31 antibody. Bright-yellow fluorescence was a composite image of red and green fluorescence, and used to indicate co-localization of liposomes with glioblastoma neovasculatures. White line was used to indicate the boundary between normal tissue and glioblastoma region in the brain. a. Blank control; b. DiI liposomes; c. Glu-targeting DiI liposomes; d. cRGD-targeting DiI liposomes; e. Functional targeting DiI liposomes.

Fig. 8A and Fig. 8B show the *in vivo* co-localization or targeted binding of varying formulations with brain glioblastoma neovasculature and with brain glioblastoma cells, respectively. The nuclei around the brain glioblastoma region are indicated by blue fluorescence by staining them with Hoechst 33342, and the brain glioblastoma neovasculature are shown as red fluorescent structures by staining with an anti-CD31 antibody. Dil-labeled liposomes are observed as green fluorescence. Bright yellow fluorescence is a composite of red and green fluorescence and is used to indicate the co-localization or targeting binding of DiI-labeled liposomes with glioblastoma neovasculatures. The co-localization or targeting binding of DiI-labeled liposomes with brain glioblastoma cells are observed by the green fluorescence in the brain glioblastoma region in an area of high-density blue fluorescence.

The results demonstrated that the functional targeting Dil liposomes were the most evidently co-localized with the brain glioblastoma neovasculatures (Fig. 8A) and with the brain glioblastoma cells (Fig. 8B) compared to the control formulations. Moreover, cRGD-targeting DiI liposomes were bound with the glioblastoma neovasculature, and cRGD-targeting DiI liposomes were bound with the glioblastoma cells. Furthermore, these co-localizations were less frequently found in normal brain tissue (Fig. 8B).

### *In vivo* imaging of the distribution in brain glioblastoma-bearing mice

**Figure 9:**
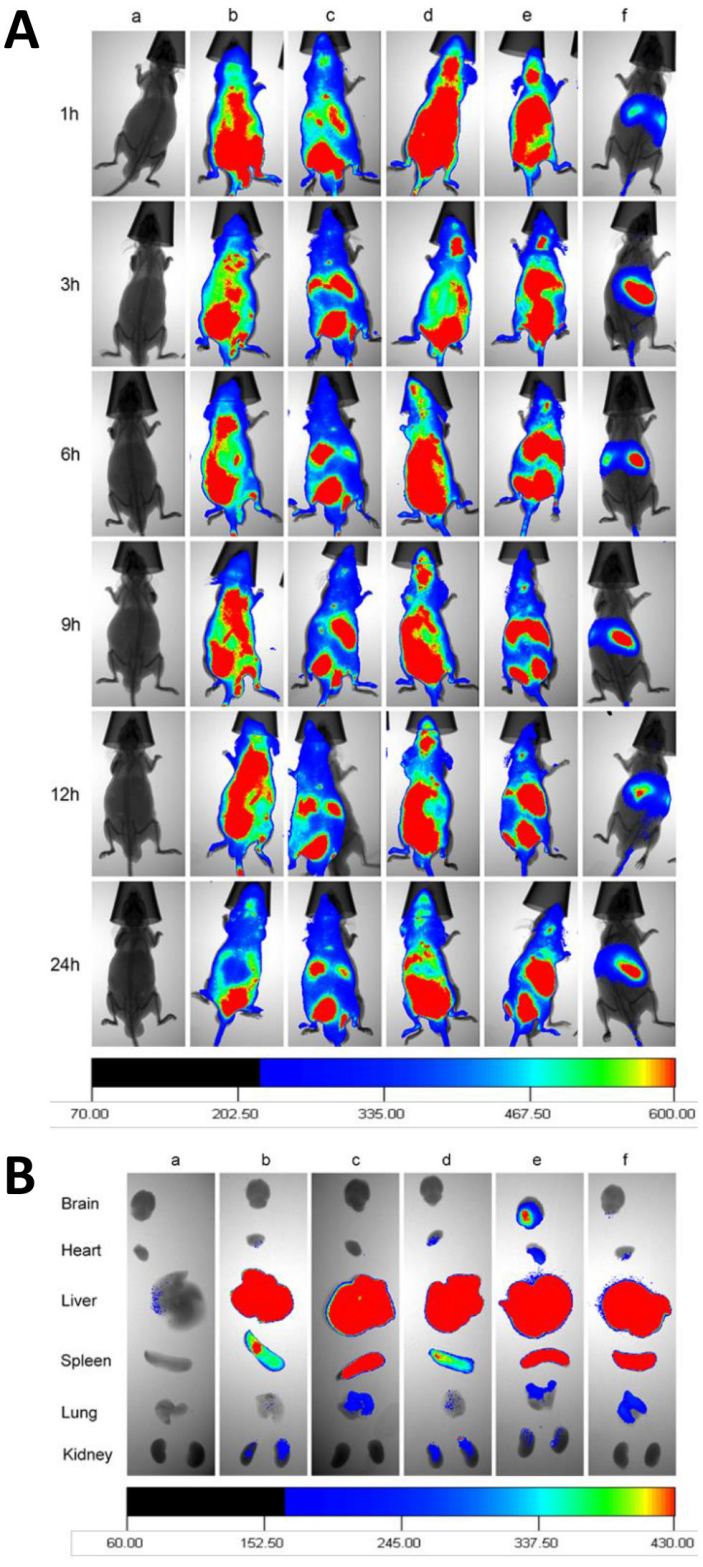
*In vivo* imaging the distribution in glioblastoma-bearing mice Notes: **A.** *In vivo* real-time image for the distribution of functional targeting DiR liposomes. **B.** *Ex vivo* images for the glioblastoma-bearing brain masses arising from glioblastoma U251 cells, and for organs after the glioblastoma-bearing male nude mice sacrificed at 72 h. a. Physiological saline; b. DiR liposomes; c. cRGD-targeting DiR liposomes; d. Glu-targeting DiR liposomes; e. Functional targeting DiR liposomes; f. free DiR.

Fig. 9 demonstrates *in vivo* real-time imaging to observe the distribution of varying fluorescent probe DiR-labeled liposomes in brain glioblastoma-bearing mice. After intravenous administration of functional targeting DiR liposomes or Glu-targeting DiR liposomes, a strong DiR fluorescent signal was observed in the brain location for up to 24 h. After intravenous administration of DiR liposomes or RGD-targeting DiR liposomes, the DiR fluorescent signal was observed in the brain location at 1 h and then gradually decreased (Fig. 9A).

After intravenous administrations of varying DiR-labeled liposomes, mice were sacrificed at 72 h, and the major organs were isolated for *ex vivo* imaging of the distribution of the liposomes. Among varying formulations, only a strong fluorescent signal of functional targeting DiR liposomes was observed in the brain tissue (Fig. 9B). In addition, fluorescent signals of all formulations at 72 h were mainly distributed in liver and spleen tissues and sparsely distributed in heart, lung, and kidney tissues.

### Overall anticancer efficacy in brain glioblastoma-bearing mice

**Figure 10:**
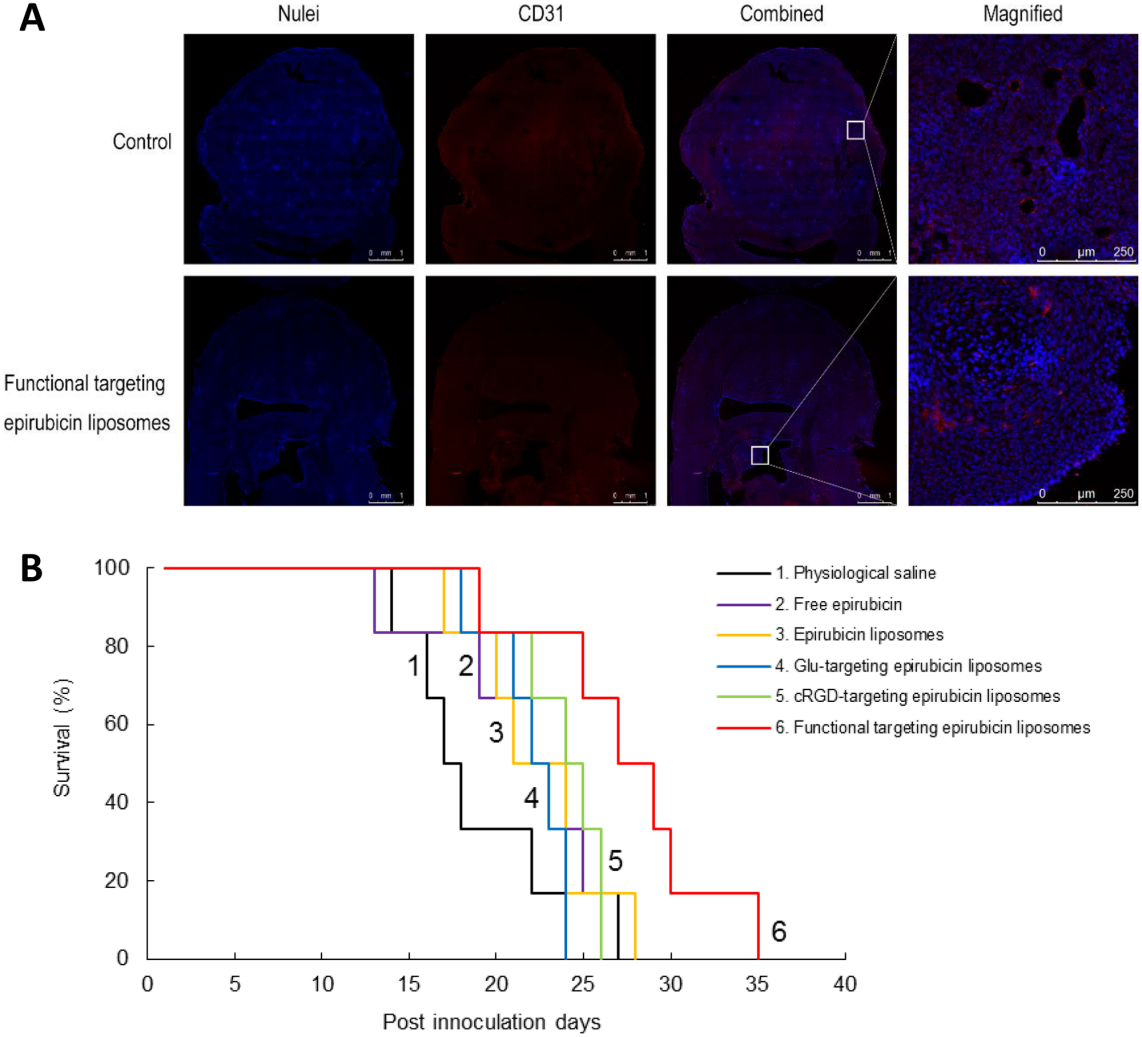
Anticancer efficacy in glioblastoma-bearing mice Notes: **A.** Destroying effect on the neovasculatures in intracranial glioblastoma-bearing nude mice. White square was the field magnified for observing morphologic change of glioblastoma neovasculatures before and after treatment with functional targeting epirubicin liposomes. **B.** Kaplan–Meier survival curves of intracranial glioblastoma-bearing nude mice treated with varying liposomes (*n* = 6).

Fig. 10 shows the destruction of neovasculatures and the anticancer efficacy of varying formulations in brain glioblastoma-bearing mice. To evaluate the destruction effects on the glioblastoma neovasculature *in vivo*, frozen brain slices were prepared from brain glioblastoma-bearing mice scarified at day 20 after multiple intravenous administrations of physiological saline or functional targeting epirubicin liposomes on days 11, 14, 16, and 18. The brain nuclei and brain glioblastoma neovasculatures in frozen slices of brain are indicated as blue and red fluorescent structures, respectively. After treatment with functional targeting epirubicin liposomes, brain neovasculatures in the glioblastoma region in mice were significantly more destroyed in relation to their quantity and morphology compared to the control. The brain glioblastoma area indicated by the nuclei was also reduced (Fig. 10A).

The Kaplan-Meier survival curves were used to evaluate the efficacy after treatment with functional targeting epirubicin liposomes (Fig. 10B). The results showed that the median survival times of brain glioblastoma-bearing mice were 17.5, 23, 22.5, 24.5, 22.5 and 28.0 days after treatment with physiological saline, free epirubicin, epirubicin liposomes, cRGD-targeting epirubicin liposomes, Glu-targeting epirubicin liposomes, and functional targeting epirubicin liposomes, respectively. Statistical analysis demonstrated that the treatment with functional targeting epirubicin liposomes significantly extended the survival of glioblastoma-bearing mice compared to the treatment with physiological saline (*p* = 0.0147), free epirubicin (*p* = 0.0205), epirubicin liposomes (*p* = 0.0463), cRGD-targeting epirubicin liposomes (*p* = 0.0435) or Glu-targeting epirubicin liposomes (*p* = 0.0118).

## DISCUSSION

The difficulties that exist in the treatment of brain cancer may arise from three major aspects: the incomplete removal of malignant cells and the complexity of surgical operations on the brain, the limited transport of anticancer drugs across the BBB, and the limited efficacy of radiation therapy on cancer cells in the deep core of the brain. Accordingly, even after a combined therapy strategy, residual malignant cells could regenerate with the support of biological nutrients via brain blood vessels and residual neovasculature in the brain cancer microenvironment. Therefore, an efficient chemotherapy that could transfer drugs across the BBB, eliminate residual malignant cells, and destroy residual neovasculature in the brain cancer region would be beneficial for overall treatment efficacy.

In the present study, functional targeting epirubicin liposomes were formulated by modifying two functional lipid-conjugate ligands, DSPE-PEG_2000_-Glu and DSPE-PEG_2000_-cRGD, to reach this goal. A glucose derivative (4-aminophenyl β-D-glucopyranoside, Glu) and a cyclic pentapeptide (c(RGDfK)mpa) were used as the targeting ligands for guiding the transport of epirubicin-loaded liposome vesicles across the BBB, followed by targeting of those vesicles to brain cancer cells and to the neovasculature around the brain cancer region. Two ligands were conjugated with DSPE-PEG_2000_ because DSPE-PEG_2000_ had been used as a long-circulating material to avoid rapid uptake by the reticuloendothelial system (RES) [27].

The functional targeting epirubicin liposomes possess a high encapsulation efficiency for drugs (>95%), suitable particle size (approximately 109 nm), and less leakage in the blood component-containing system. These properties allow the liposomes to be stable in blood circulation and to be more highly accumulated around the cancer tissue through the enhanced permeability retention (EPR) effect [28].

In the cytotoxicity assay, the efficacy of functional targeting epirubicin liposomes against brain glioblastoma was shown to be effective at a low concentration of epirubicin (<0.6 μM). Compared with the control formulations, the IC_50_ value was also reduced. These experiments demonstrate the *in vitro* efficacy of the newly developed formulation in the treatment of brain glioblastoma and exhibit a therapeutic advantage over regular drug formulations such as free epirubicin injections.

The targeting effects of functional targeting coumarin liposomes were defined in two ways: targeting the BBB and targeting brain glioblastoma cells (or neovasculature). To observe the targeting effect on the BBB, BMVECs were included for investigation by studying the targeted binding with Glut-1, while the transport ability across the BBB was a result of the targeted binding discussed below. To understand the targeting effect on brain glioblastoma cells, the binding of functional targeting coumarin liposomes with integrin β3 receptors on glioblastoma cells was included for observation. Actually, integrin β3 receptors are also overexpressed on the neovasculatures of brain glioblastoma [3]. Therefore, the targeting effect by binding with integrin β3 receptors also reflects the potential targeting effect on the neovasculatures. The results demonstrate that the binding with Glut-1 facilitates the active targeting of functional targeting coumarin liposomes or Glu-targeting coumarin liposomes to the BMVECs, while binding with integrin β3 receptors promotes the internalization of functional targeting epirubicin liposomes or cRGD-targeting epirubicin liposomes into glioblastoma cells. The results from the fluorescence probe coumarin-labeled functional targeting liposomes indicate obvious co-localization with Glut-1 and integrin β3 receptors and show that the dual modifications of two targeting conjugates do not interfere with each other in the binding or targeting effects.

Results from the uptake ratios performed on BMVECs and on brain glioblastoma cells further revealed the targeting effects, showing evidently enhanced uptake ratios in two types of cells after treatment with the functional targeting coumarin (or epirubicin) liposomes compared to treatment with the control formulations. These facts were further confirmed by the competition inhibition experiments of cellular uptake, which demonstrated that the enhanced uptake of DSPE-PEG_2000_-Glu modified liposomes in BMVECs was effectively inhibited by adding excess glucose and that the enhanced uptake of DSPE-PEG_2000_-cRGD modified liposomes in glioblastoma cells was lowered by adding excess free c(RGDfK)mpa.

To mimic the transport of functional targeting epirubicin liposomes across the BBB and the targeted killing effect on brain glioblastoma, the co-culture BBB model was successfully built by culturing BMVECs with astrocytes in the upper insert, and by culturing glioblastoma cells in the lower compartment in the well. Astrocytes stimulate the formation of the BBB by secreting glia-derived factors such as transforming growth factor-β (TGF-β), glial-derived neurotrophic factor (GDNF) [29, 30], basic fibroblast growth factor (bFGF) and angiopoietin-1 [31] to nourish the BMVECs. The BBB membrane was formed by the tight junction of BMVECs, and the integrity of the membrane was observed by monitoring the TEER values [32] and the leakage of liquid, indicating the suitability of the experiment for transport across the BBB. The results elucidate the ability of functional targeting epirubicin liposomes to be transported across the BBB and the killing efficacy for the treatment of glioblastoma cells. Among multiple control groups, functional targeting epirubicin liposomes or Glu-targeting epirubicin liposomes exhibited higher transport ratios across the BBB, indicating that Glut-1 is acting as a transporting ferry across the BBB via a carrier-mediated transcytosis pathway [33, 34]. Furthermore, functional targeting epirubicin liposomes exhibited the strongest killing effect on glioblastoma cells. The likely reason also derives from the targeting action via the binding of cRGD on the functional targeting epirubicin liposomes with integrin β3 receptors overexpressed on the brain glioblastoma cells.

Multicellular cancer spheroids reflect aspects of solid cancer characteristics *in vivo*, such as intimate cell-cell contact [35]. In this study, the multicellular cancer spheroids were established with glioblastoma U251 cells to evaluate the cancer-penetrating ability of functional targeting epirubicin liposomes. The results reveal that functional targeting epirubicin liposomes had the strongest penetrating ability in glioblastoma spheroids compared to the control formulations. Previous studies have shown that both integrin β3 receptors and Glut-1 are overexpressed on brain tumor cells [36]. Therefore, the strongest penetrating ability of functional targeting epirubicin liposomes may be explained by the dual-targeting effects via integrin β3 receptor-mediated endocytosis and Glut-1-mediated transcytosis in glioblastoma spheroids.

Human umbilical vein endothelial cells (EA. hy 926) were chosen to mimic the neovasculature in the brain glioblastoma region for investigating the antiangiogenic effect of functional targeting epirubicin liposomes. The measured IC_50_ values indicate that the functional targeting epirubicin liposomes have the strongest inhibitory effect on the endothelial cells. Moreover, the microscope images and quantitative results from the 3D Matrigel model established with endothelial cells (EA. hy 926) further reveal the most significant destroying effect of functional targeting epirubicin liposomes on neovasculature compared to that of epirubicin liposomes or Glu-targeting epirubicin liposomes. In addition, cRGD-targeting epirubicin liposomes show a stronger disabling effect on the neovasculature in glioblastoma. The possible mechanism may be related to the increased internalization of cRGD-modified targeting epirubicin liposomes in endothelial cells, which overexpress integrin β3 receptors [15], hence promoting the cytotoxic effect on endothelial cells (EA. hy 926). Studies have shown that cRGD acts as an integrin β3 antagonist and thus inhibits the migration, invasion, survival and proliferation of endothelial cells [37], while these actions encourage the growth of neovasculature in solid cancer tissues.

*In vivo* evaluations were conducted on brain glioblastoma-bearing nude mice, including documenting the targeting effect on neovasculature in brain glioblastoma, imaging the distribution, and determining overall anticancer efficacy. To directly observe the targeting binding or co-localization of functional targeting liposomes, Dil was selected as a suitable fluorescent probe to label the liposomes. The results of the confocal laser scanning fluorescent microscopic study showed clearly observed co-localizations between functional targeting DiI liposomes and brain glioblastoma neovasculatures. The same phenomenon was also observed between cRGD-targeting DiI liposomes and brain glioblastoma neovasculature, suggesting that DSPE-PEG_2000_-cRGD modified drug-loaded liposomes are able to target and bind with brain glioblastoma neovasculatures and are hence beneficial for anti-angiogenesis effects. Furthermore, cRGD-modified targeting DiI liposomes possess an evident targeting ability for brain glioblastoma cells and penetrate more deeply into brain glioblastoma tissue. The mechanism for targeted binding with glioblastoma neovasculature or with glioblastoma cells are obviously related to the targeted binding of cRGD with integrin β3 receptors [38].

For real-time imaging of the kinetic distribution of functional targeting liposomes *in vivo*, another fluorescent probe, DiR, was used to label the liposomes. The results revealed that the functional targeting DiR-labeled liposomes or Glu-targeting DiR liposomes could accumulate more into the brain tissue and stay longer compared to control formulations in brain glioblastoma-bearing mice. In contrast, free DiR, DiR liposomes, and cRGD-targeting DiR liposomes were mainly distributed in liver and spleen tissues and were rapidly eliminated from mice. These results also provided evidence for the targeted accumulation of functional targeting liposomes in brain glioblastoma tissue.

To observe the destruction of neovasculature and the overall anticancer efficacy in brain glioblastoma-bearing mice, an immunofluorescence technique and survival curve plotting were performed on the brain glioblastoma-bearing mice. The results demonstrated that the functional targeting epirubicin liposomes are able to dramatically destroy brain glioblastoma neovasculature and to extend the survival of brain glioblastoma-bearing mice. As the nude mice have shorter lifetime compared with human beings, the extended lifetime of the glioblastoma-bearing mice after treatment could not be a very long time, despite there exists a statistically difference between different treatment groups. In addition, only one human brain glioblastoma cell line is used in this study, it is expected that the functional targeting epirubicin liposomes could exhibit anti-cancer effect in more patient-derived brain glioma cells, which will be subject to future investigations. Nevertheless, the *in vivo* data demonstrate the promising positive outcomes that the functional targeting epirubicin liposomes are able to transfer across the BBB, target glioblastoma cells, and disable neovascularization in brain glioblastoma-bearing mice.

## MATERIALS AND METHODS

### Materials and cells

Epirubicin hydrochloride was purchased from Nanjing Tianzun Zezhong Chemicals, Co. Ltd. (Nanjing, China). Egg phosphatidylcholine (EPC) was purchased from Lipoid GmbH (Ludwigshafen, Germany). Cholesterol was purchased from J&K Scientific, Ltd. (Beijing, China). DSPE-PEG_2000_, DSPE-PEG_2000_-succinimide (DSPE-PEG_2000_-NHS) and DSPE-PEG_2000_-maleimide were obtained from the NOF Corporation (Tokyo, Japan). Cyclo Arg-Gly-Asp-D-Phe-Lys mercaptopropionic acid (c(RGDfK)mpa) was synthesized by GL Biochem, Ltd. (Shanghai, China). 4-Aminophenyl β-D-glucopyranoside was purchased from Sigma Aldrich (St. Louis, MO, USA). Other chemicals were analytical or HPLC grade.

Human glioblastoma U251 cells and human umbilical vein endothelial cell (EA. hy 926) were purchased from the Cell Resource Center of Peking Union Medical College (Beijing, China). Murine brain microvascular endothelial cells (BMVECs) were obtained from the Institute of Clinical Medical Sciences at China-Japan Friendship Hospital (Beijing, China). Rat astrocytes (RAs) and their culture medium were purchased from ScienCell Research Laboratories (Carlsbad, CA, USA). All cells were cultured in an incubator containing 5% CO_2_ at 37°C. All culture media and growth factors were purchased from Macgene Biotech, Co., Ltd. (Beijing, China) unless otherwise noted. The culture medium for the glioblastoma U251 cells was Eagle's minimum essential medium with Earle's balanced salts (MEM-EBSS) supplemented with 10% fetal bovine serum (FBS; Gibco, Carlsbad, California, USA), and the medium for BMVECs was endothelial cell culture medium (Dulbecco's modified Eagle's medium, DMEM, 20% FBS, 100 mg/mL endothelial cell growth factor, 40 U/mL heparin). The culture medium for EA. hy 926 endothelial cells was DMEM supplemented with 10% FBS. The RAs were cultured in an astrocyte medium (ScienCell, USA). Male BALB/C nude mice (18–22 g) were obtained from the Peking University Experimental Animal Center (Beijing, China).

### Synthesis of targeting molecules

DSPE-PEG_2000_-Glu conjugate was newly developed by conjugating DSPE-PEG_2000_-NHS with 4-aminophenyl β-D-glucopyranoside. Briefly, 4-aminophenyl β-D-glucopyranoside and DSPE-PEG_2000_-NHS were dissolved at a ratio of 1:1 (w/w) in anhydrous N,N-dimethylformamide (DMF), and the coupling reaction was carried out at room temperature under argon protection in a light-resistant container for 48 h. Then, the reaction mixture was transferred into dialysis tubing (molecular-weight cutoff, MWCO, 1000 Da) and dialyzed against deionized water for 36 h to remove the DMF solvent and uncoupled molecules. The resultant was then freeze dried and stored at −20°C. The conjugation of DSPE-PEG_2000_-Glu was confirmed by proton nuclear magnetic resonance spectroscopy (400 MHz ^1^H NMR, Bruker AVANCE III 400).

The DSPE-PEG_2000_-cRGD conjugate was synthesized from DSPE-PEG_2000_-maleimide and c(RGDfK)mpa. Briefly, c(RGDfK)mpa and DSPE-PEG_2000_-maleimide were dissolved at a ratio of 3:1 (mol/mol) in phosphate buffered saline (137 mM NaCl, 2.7 mM KCl, 8 mM Na_2_HPO_4_ and 2 mM KH_2_PO_4_, PBS pH 7.4), and the coupling reaction was carried out at room temperature for 48 h. The reaction mixture was then transferred into dialysis tubing (MWCO, 1000 Da) and dialyzed against deionized water for 36 h to remove the uncoupled molecules. The resultant was freeze dried and stored at −20°C. The conjugation of DSPE-PEG_2000_-cRGD was confirmed by matrix-assisted laser desorption/ionization time of flight mass spectrometry (MALDI-TOF-MS, Shimadzu, Japan).

### Formulation and characterization of the liposomes

To prepare blank functional targeting liposomes, egg phosphatidylcholine (EPC), cholesterol, DSPE-PEG_2000_-cRGD and DSPE-PEG_2000_-Glu were mixed in chloroform at a ratio of 60:34:3:3 (mol/mol) in a pear-shaped bottle. The solvent was removed with a rotary vacuum evaporator at an elevated temperature (50°C), and the lipid film was hydrated with 250 mM ammonium sulfate by sonication in a water bath for 5 min. Subsequently, the liposomes were sonicated at 220 W for 10 min using an ultrasonic cell disruptor (Scientz Biotechnology Co., Ltd., Ningbo, China). The liposomes obtained were extruded through polycarbonate membranes (Millipore, Bedford, MA, USA) three times with 400 nm pores and then three times with 200 nm pores.

To prepare functional targeting epirubicin liposomes, the above blank liposomes were dialyzed (MWCO, 12,000–14,000 Da) in PBS (pH 7.4) for 24 h and incubated with epirubicin solution at 60°C with continual shaking for 40 min (lipids:drug 30:1, w/w). The functional targeting epirubicin liposomes were then obtained.

cRGD-targeting epirubicin liposomes were prepared by replacing DSPE-PEG_2000_-Glu with DSPE-PEG_2000_. Glu-targeting epirubicin liposomes were prepared by replacing DSPE-PEG_2000_-cRGD with DSPE-PEG_2000_. Epirubicin liposomes were prepared using DSPE-PEG_2000_ to substitute for DSPE-PEG_2000_-cRGD and DSPE-PEG_2000_-Glu.

In addition, three types of liposomes were similarly prepared as fluorescent probes for evaluating cellular uptake and targeting effects, including coumarin-labeled liposomes (lipids:coumarin 6 = 500:1, w/w), DiR-labeled liposomes (lipids:DiR = 200:1, w/w) and DiI-labeled liposomes (lipids:DiI = 200:1, w/w).

The particle sizes, polydispersity index (PDI) and zeta potential values were measured with a Nano Series Zen 4003 Zetasizer (Malvern, UK). Each assay was repeated three times. Atomic force microscopy (AFM, SPI3800N series SPA-400, NSK Ltd., Tokyo, Japan) and transmission electron microscopy (TEM, Tecnai G2 20ST, FEI Co., Japan) were used to further observe the morphology of the liposomes.

The encapsulation efficiency (EE) of epirubicin was calculated using the formula: EE = (W_encap_/W_total_) × 100%, where W_encap_ is the measured amount of epirubicin in the liposome suspensions after passing over the Sephadex G-50 column, and W_total_ is the measured amount of epirubicin in an equal volume of initial liposome suspensions. *In vitro* release of epirubicin from the liposomes was performed by dialyzing against release medium containing serum protein (pH 7.4 PBS containing 10% FBS) at 37°C. The release rate (RR, %) was calculated using the formula: RR = (W_i_/W_total_) × 100%, where W_i_ is the measured amount of epirubicin at the i^th^ time-point in the release medium, and W_total_ is the total amount of epirubicin in an equal volume of liposome suspensions prior to dialysis. The liposomes used in the experiments were disrupted by methanol. The concentration of epirubicin was analyzed using the high-performance liquid chromatography method (Agilent Technologies Inc. Cotati, CA, US) at a UV wavelength of 254 nm with a mobile phase consisting of acetonitrile, 0.02 M NaH_2_PO_4_, and triethylamine (34:66:0.3, v/v/v, pH 4.0 adjusted with acetic acid). The analysis was performed on an ODS column (ZORBAX Extend-C18, 4.6 × 250 mm, Agilent, USA) at a flow rate of 1.0 mL/min. Each assay was repeated three times.

### Cytotoxicity in glioblastoma cells

To evaluate the cytotoxic effects of varying liposomes, glioblastoma U251 cells were seeded at a density of 5000 cells/well in 96-well culture plates and cultured for 24 h. Then, the cells were exposed to free epirubicin, epirubicin liposomes, cRGD-targeting epirubicin liposomes, Glu-targeting epirubicin liposomes and functional targeting epirubicin liposomes. The concentrations of epirubicin were in the range of 0–5.0 μM. After incubation for 48 h, the cytotoxic effects were evaluated with a sulforhodamine B (SRB) colorimetric assay. Briefly, the culture medium was removed, and then, the cells were fixed with trichloroacetic acid followed by washing with deionized water and staining with SRB. Measurement was performed at 540 nm using a microplate reader (Tecan Infinite F50, Tecan Group Ltd., Shanghai, China). The survival rates of glioblastoma cells were calculated using the following formula: survival % = (A_540 nm_ for treated cells / A_540 nm_ for control cells) × 100%, where A_540 nm_ represents the absorbance value. GraphPad Prism 6 software (GraphPad Software, Inc., California, USA) was used to calculate the IC_50_ value.

### Targeting effects on BMVECs and on glioblastoma cells

To observe the targeting effects, BMVECs and glioblastoma U251 cells were seeded into chambered coverslips at a density of 1.5 × 10^5^ cells/well, respectively. The culture medium for BMVECs was maintained in low-glucose DMEM culture medium (Macgene, China) in this experiment to reduce the glucose competition effect. After incubation for 24 h, the BMVECs and glioblastoma U251 cells were treated with coumarin liposomes, cRGD-targeting coumarin liposomes, Glu-targeting coumarin liposomes and functional targeting coumarin liposomes at a final concentration of 1 μM coumarin for another 3 h. After incubation with different types of liposomes, BMVECs and glioblastoma U251 cells were fixed with 4% paraformaldehyde at 25°C for 10 min, and then treated with PBS-containing goat serum (PBS pH 7.4, 10% goat serum, 0.3 M glycine) at 4°C for 5 h to block nonspecific binding of the antibodies in the following experimental steps. After being washed with PBS (pH 7.4), BMVECs and glioblastoma U251 cells were separately stained with a rabbit anti-glucose transporter Glut-1 antibody (1:250 dilution, Abcam, Cambridge, UK) and a rabbit anti-integrin β3 antibody (1:100 dilution, Abcam) at 4°C for 16 h. After being washed with PBST solution (PBS pH 7.4, 0.2% Tween-20, v/v) three times, the cells were labeled with Alexa Fluor 647-conjugated goat anti-rabbit IgG H&L (1:500 dilution, Abcam) at room temperature for 1 h. Then, the cells were washed three times with PBST solution, and the nuclei of the cells were stained with 2 μg/mL Hoechst 33342 for 10 min. Finally, the cells were imaged and analyzed with a confocal laser scanning fluorescent microscope (Leica, Heidelberg, Germany). The excitation and emission wavelengths of coumarin were set at 488 nm and 505 nm, respectively.

### Uptake and mechanism in BMVECs and glioblastoma cells

Coumarin-labeled liposomes and epirubicin-loaded liposomes were used as fluorescent probes for observing cellular uptake by BMVECs and by glioblastoma cells, respectively. To study cellular uptake, BMVECs (maintained in low-glucose DMEM) or glioblastoma U251 cells were seeded into six-well plates at a density of 2 × 10^5^ cells/well and cultured for 24 h. Afterward, BMVECs were incubated with coumarin liposomes, cRGD-targeting coumarin liposomes, Glu-targeting coumarin liposomes or functional coumarin liposomes at a final concentration of 1 μM coumarin, while glioblastoma U251 cells were incubated with epirubicin liposomes, cRGD-targeting epirubicin liposomes, Glu-targeting epirubicin liposomes or functional targeting epirubicin liposomes at a final concentration of 15 μM epirubicin. Drug-free culture medium was used as the blank control for each. After further incubation for 3 h, the cells were washed three times with cold PBS (pH 7.4) and then re-suspended in 400 μl PBS (pH 7.4). Coumarin fluorescence intensity or epirubicin fluorescence intensity was measured with a flow cytometer (Becton Dickinson FACSCalibur, Mountain View, USA).

To further investigate the cellular uptake mechanism of functional liposomes, competition inhibition was conducted. Briefly, BMVECs (maintained in low-glucose DMEM) or glioblastoma U251 cells were seeded into chambered coverslips at a density of 1.5 × 10^5^ cells/well. After incubation for 24 h, a concentrated free glucose solution was added to the BMVECs to reach a final concentration of 500 μM glucose in each well and incubated for 1 h. Coumarin-labeled liposomes were then added at a final concentration of 1 μM coumarin and further incubated for 2 h. Similarly, a concentrated free c(RGDfK)mpa solution was added to the glioblastoma U251 cells to reach a final concentration of 100 μM free c(RGDfK)mpa and incubated for 1 h. Afterward, epirubicin liposomes, cRGD-targeting epirubicin liposomes, Glu-targeting epirubicin liposomes and functional targeting epirubicin liposomes were separately added at a final concentration of 15 μM epirubicin and further incubated for 2 h. After being washed with PBS (pH 7.4), BMVECs or glioblastoma U251 cells were stained with 4 μg/mL Hoechst 33342 for 10 min. Finally, the cells were imaged and analyzed with a confocal laser scanning fluorescent microscope. The excitation and emission wavelengths for measuring coumarin were set at 488 nm and 505 nm, respectively. The excitation wavelength for measuring epirubicin was set at 488 nm while the emission wavelength was in the range of 575–585 nm.

### Transporting across the BBB and treating glioblastoma cells

To assess the ability of functional targeting epirubicin liposomes to transport across the BBB and to kill brain cancer cells, a co-culture BBB model was developed with BMVECs/glioblastoma cells, according to a previous report [39] with modification. Briefly, astrocytes were seeded in the bottom of the well at 1.5 × 10^5^ cells/well in 12-well plates 2 days before the endothelial cells were seeded into the upper insert. To facilitate astrocyte adhesion, the well bottoms were treated with 5 μg/cm^2^ poly-D-lysine for at least 12 h at 37°C before seeding. Then, the BMVECs were seeded onto the membrane of the upper insert at 3.75 × 10^4^ cells/insert and grew together with RAs for another 5 days. To facilitate BMVEC adhesion, the membrane was coated with gelatin (2%, w/v, D-Hank's buffer solution) for at least 1 h in the culture incubator before the BMVECs were seeded. At day 7, the upper insert was transferred to another 12-well culture plate where glioblastoma U251 cells had already been cultured in the bottom of the well at 3.75 × 10^4^ cells/insert for 1 day. After incubation for another day, the co-culture BBB model containing BMVECs/glioblastoma cells was established and ready for experiments.

To assess the ability of functional epirubicin liposomes to be transported across the BBB, the transport ratio (TR) of epirubicin was measured using the formula: TR = (W_3_/W_total_) × 100%, where W_3_ is the measured amount of epirubicin at 3 h in the bottom well medium, and W_total_ is the total amount of epirubicin in an equal volume of free epirubicin or liposome suspensions prior to dialysis. Different formulations were added into the upper insert, including free epirubicin, epirubicin liposomes, cRGD-targeting epirubicin liposomes, Glu-targeting epirubicin liposomes and functional targeting epirubicin liposomes. The final concentration of epirubicin was 5 μM for each formulation. After 3 h, epirubicin in the bottom well was determined by the HPLC method as above.

To investigate the killing effects after transporting across the BBB, the above formulations were similarly added into the upper insert of the co-culture BBB model. The final concentration of epirubicin was 5 μM for each formulation. After 3 h, the medium in the upper insert was changed to a fresh BMVECs culture medium. After incubation for another 48 h, the percentage of surviving glioblastoma U251 cells in the lower layer of plate well was determined by the SRB staining assay.

### Penetrating into solid glioblastoma spheroids

Multicellular brain cancer spheroids were established with glioblastoma U251 cells to evaluate the penetrating ability of varying liposomes into solid glioblastoma spheroids. Briefly, agarose was added into a serum-free MEM-EBSS culture medium and heated to 80°C for 30 min to form a 2% (w/v) solution. Each well of the 96-well culture plates was coated with a thin layer (50 μl) of the sterilized agarose solution. After cooling to room temperature, glioblastoma U251 cells were seeded with 100 μl growth medium at 1 × 10^3^ cells/well. The culture plates were gently shaken for 3 min to balance the cancer cells and incubated at 37°C for 7 days to form solid brain cancer spheroids.

To evaluate the penetrating ability, coumarin-labeled liposomes were used as fluorescent probes. Briefly, glioblastoma spheroids were treated with free coumarin, coumarin liposomes, cRGD-targeting coumarin liposomes, Glu-targeting coumarin liposomes or functional coumarin liposomes at a concentration of 1 μM coumarin. After incubation for 12 h, the glioblastoma spheroids were then washed with PBS (pH 7.4), and the different layers were scanned from the top to the bottom of the spheroids using a confocal laser scanning fluorescent microscope.

### Inhibiting endothelial cells and destroying neovasculatures

In mimicking the inhibitory effects of varying liposomes on blood vessels in the brain glioblastoma region, endothelial cells (EA. hy 926) were seeded at a density of 5000 cells/well in 96-well culture plates and cultured for 24 h. Then, the cells were exposed to free epirubicin, epirubicin liposomes, cRGD-targeting epirubicin liposomes, Glu-targeting epirubicin liposomes or functional targeting epirubicin liposomes. The concentrations of epirubicin were in the range of 0–5.0 μM. After incubation for another 48 h, the destroying effects were observed with an SRB colorimetric assay. GraphPad Prism 6 software was used to calculate the IC_50_ value.

The destructive effect on the neovasculature was assessed using a three-dimensional (3D) Matrigel system established with endothelial cells (EA. hy 926). Briefly, a 96-well culture plate was coated with Matrigel (50 μL/well, BD Biosciences, USA) at 37°C for 30 min. Endothelial cells (EA. hy 926) were seeded at 1 × 10^4^ cells/well, and then free epirubicin, epirubicin liposomes, cRGD-targeting epirubicin liposomes, Glu-targeting epirubicin liposomes or functional targeting epirubicin liposomes were added into the culture system at a concentration of 20.0 μM epirubicin. Drug-free culture medium was used as the blank control. After drug treatment, the cells were incubated for 12 h. Tube formation was observed and quantified by measuring the number of endothelial cell loops (capillary-like structures) in three random visual fields with two-dimensional microscope images of the culture dish (Caikon Optical Instrument Co., Ltd., Shanghai, China).

### Co-localization with neovasculature and with cancer cells in brain glioblastoma-bearing mice

To build a brain cancer-bearing model, male BALB/c nude mice (18–20 g) were included in the study. All procedures were performed according to the guidelines of the Institutional Authority for Laboratory Animal Care of Peking University. For intracranial glioblastoma implantation, nude mice were anesthetized and fixed in a stereotactic device. An incision was made, and a burr hole was drilled to a position 1.5 mm posterior and 1.8 mm lateral to the bregma in the right cerebral hemisphere. Glioblastoma U251 cells (6 × 10^5^ cells/3 μl, MEM-EBSS culture medium) were injected into the right striatum of the brain to a depth of 3 mm at a rate of 1 μl/min. The craniotomy was covered with bone wax, and the incision was closed with sutures.

To evaluate the *in vivo* targeting property of varying liposomes to the neovasculature and to brain cancer cells, the targeting co-localizations were observed using immunofluorescence staining followed by confocal laser scanning fluorescent microscopy. Briefly, brain glioblastoma-bearing mice were randomly divided into five groups (3 per group). At day 14 post-inoculation of glioblastoma, varying fluorescent probe DiI-labeled liposomes were injected into the glioblastoma-bearing nude mice through the tail vein, including DiI liposomes, cRGD-targeting DiI liposomes, Glu-targeting DiI liposomes and functional targeting DiI liposomes, at a dose of 100 μg/kg for each animal. Physiological saline was administered as a blank control. After 2 h, the nude mice were anesthetized, and the hearts were perfused with physiological saline followed by perfusion with 4% paraformaldehyde. The brains were removed for the preparation of consecutive frozen slices of 20 μm thickness. Glioblastoma neovasculatures were stained with a goat anti-CD31 antibody (1:300 dilution, Abcam) followed by an Alexa Fluor 647-conjugated goat anti-rabbit IgG H&L (1:500 dilution, Abcam). The nuclei of the cells were stained with 2 μg/mL Hoechst 33342 for 15 min. The fluorescence was observed with a confocal laser scanning fluorescent microscope. The excitation and emission wavelengths of DiI were set at 549 nm and 565 nm, respectively.

### *In vivo* imaging of the distribution in brain glioblastoma-bearing mice

Noninvasive optical imaging systems were used to observe the real-time distribution and tumor accumulation ability of DiR-labeled liposomes in glioblastoma-bearing nude mice. The lipophilic fluorescent DiR dye was encapsulated in the membrane of the liposomes and used as a probe to indicate the distribution of the liposomal formulation in brain glioblastoma-bearing mice. At day 14 after the intracranial implantation of glioblastoma U251 cells, the nude mice were divided into six groups (3 per group) and were administered via the tail vein free DiR, DiR liposomes, cRGD-targeting DiR liposomes, Glu-targeting DiR liposomes or functional targeting DiR liposomes at a dose of 100 μg/kg for each animal. Physiological saline was administered as a blank control. Then, the nude mice were scanned at various time points (1, 3, 6, 9, 12 and 24 h) using a Kodak multimodal imaging system (Carestream Health, Inc., USA).

To further observe the distribution status in tumor masses and the major organs, the brain glioblastoma-bearing mice were sacrificed at 72 h, followed by the immediate removal of the brain, heart, liver, spleen, lung, and kidney. The fluorescence signal intensities in different organs were photographed using a Kodak multimodal imaging system.

### Overall anticancer efficacy in brain glioblastoma-bearing mice

The overall anticancer efficacy was evaluated by observing the efficacy of destroying neovasculature *in vivo* and by monitoring the survival status in brain glioma-bearing mice. Briefly, the brain glioblastoma-bearing nude mice were randomly divided into six groups (9 per group). At days 11, 14, 16 and 18 post-inoculation of glioblastoma cells, free epirubicin, epirubicin liposomes, cRGD-targeting epirubicin liposomes, Glu-targeting epirubicin liposomes or the functional targeting epirubicin liposomes were administered to mice via the tail vein at a dose of 5 mg/kg epirubicin for each animal. Physiological saline was administered as a blank control. The frozen slices of brain tissue after treatment with functional targeting epirubicin liposomes (at day 20) were used to evaluate the destruction of the neovasculature in intracranial glioblastoma-bearing nude mice (3 mice per group).

The tumor neovasculature was stained with a goat anti-CD31 antibody (1:300 dilution, Abcam) followed by Alexa Fluor 647-conjugated goat anti-rabbit IgG H&L (1:500 dilution, Abcam) using the same method described above. The nuclei of the cells were stained with 2 μg/mL Hoechst 33342 for 10 min. The distribution of fluorescence was observed with a confocal laser scanning fluorescent microscope.

The remaining 6 mice in each group were used for monitoring survival. Survival time was calculated from day 0 since inoculation to the day of death. Kaplan-Meier survival curves were plotted for each group. The median survival times were calculated with GraphPad Prism 6 software.

### Statistics

Data are presented as the mean ± standard deviation. One-way ANOVA was used to determine the significance among groups, after which the Bonferroni correction was used for multiple comparisons between individual groups. A value of *p* < 0.05 was considered to be significant.

## CONCLUSION

In the present study, functional targeting epirubicin liposomes were developed by modifying a new glucose-lipid conjugate DSPE-PEG_2000_-Glu and a cyclic pentapeptide-lipid conjugate DSPE-PEG_2000_-cRGD as the targeting molecules. The functional targeting epirubicin liposomes were significantly able to be transported across the BBB and showed obvious efficacies in killing glioblastoma cells and in destroying glioblastoma neovasculature *in vitro* and in brain glioblastoma-bearing nude mice. The targeting effects of functional targeting fluorescent probe-labeled liposomes on the brain tissues and on the brain glioblastoma neovasculatures are evidenced by visual observations of brain glioblastoma-bearing mice. The action mechanisms of functional targeting epirubicin liposomes are associated with the following features: the long circulation in the blood system due to the PEGylated modification of the liposomes, the ability to be transported across the BBB via the carrier-mediated transcytosis of glucose transporter-1 bound with Glu segments on the surface of the liposomes, and the targeting effects on glioblastoma cells and on the endothelial cells of glioblastoma neovasculature via the receptor-mediated endocytosis of integrin β3 receptors bound with cRGD segments on the surface of the liposomes. Therefore, the development of functional targeting epirubicin liposomes provides a potential therapy for treating brain glioblastoma and disabling neovascularization in the brain glioblastoma region.
